# Supplementary material for: GSK3β-SCFFBXW7α mediated phosphorylation and ubiquitination of IRF1 are required for its transcription-dependent turnover
Source: Nucleic Acids Res. 2019 Mar 11;47(9):4476–94. doi: 10.1093/nar/gkz163 (PMC6511875; doi:10.1093/nar/gkz163)
Supplement: Supplementary Data [file gkz163_supplemental_file.pdf]

Supplemental Figure Legends

Supplementary Figure 1. IRF1 is phosphorylated by GSK3β

- **A.** Alignment of the IRF1 GSK3 consensus site with a subset of other known GSK3 substrates, all sequences are human and the location indicated in parenthesis. Grey highlights indicate phosphorylated residues
- **B.** Schematic representation of IRF1 functional domains, indicating secondary structure within the DNA binding domain. The TAD (Transcriptional Activation Domain) and Enhancer domains are predicted to be unstructured. The location of the TPALSP motif within the transactivation domain is indicated. The conserved Thr180/181 and Ser184/185 (mouse/ human) GSK3β phosphorylation sites are highlighted in red.
- **C.** Immunoprecipitation experiments revealing phosphorylation of IRF1 T181 by GSK3β. HEK293 were transfected with expression vectors for YFP-IRF1 WT, YFP-IRF T181A, YFP-IRF1 S185A or YFP-IRF1 T181A/S185A together with GSK3β-HA vector or empty vector. Whole cell extracts were immunoprecipitated with anti-pT/S antibody and probed with anti-GFP antibody to detect YFP-IRF1 proteins, indicating T181 phosphorylation levels. The middle panel shows detection of YFP-IRF1 proteins and β-actin loading control (at 10% input for IP). The bottom panel shows GSK3β
- **D.** Quantification of increased pT/S cross-reaction with FLAG-IRF1 upon GSK3β over-expression. Data is from 8 independent experiments performed as for figure 1E. The ratio of the pT/S signal versus the FLAG-IRF1 IP (to control for IP efficiency) is shown. Statistical significance determined by Students t-test.
- **E.** Co-immunoprecipitation between IRF1 and GSK3β detected using rabbit IgG or IRF1 (C20 human specific) antibodies from H3396 cells treated with IFNγ (500U/mL) for 5 hours (plus 3 hour treatment of MG132 or DMSO) prior to lysis.

Supplementary Figure 2. GSK3β is required for IRF1 transcriptional activity

- **A.** Indirect immunofluorescence of FLAG-IRF1 and mutants in Cos7, scale bar = 10μm
- **B.** Reporter assay in MRC5 using the TRAIL-Luc reporter. Statistical differences are determined between WT and mutant IRF1.
- **C.** H3396 stable cells lines (vector, WT or T181A IRF1) treated with Dox for either 24 or 36 hours to induce expression of IRF1, β-actin is shown as loading control.
- **D.** Independent H3396 clones for each of empty vector, WT IRF1 and T181A treated as for figure 4A, the expression of IRF1 is shown below as for figure S3C.
- **E.** Chromatin immunoprecipitation of RNA-Pol II in H3396 cells treated with IFNγ (1000U/mL for 3 hr) followed by QPCR across promoter regions of indicated genes identified as containing IRF1 binding sites by ChIP-chip (1).
- **F.** As for E but using MRC5 cells.

Supplementary Figure 3. Stability of IRF1 T181A is not affected by GSK3β over-expression and GSK3β inhibition stabilises IRF1

- **A.** Data from figure 6A re-graphed as a bar chart with significance determined by Student t-test comparing % remaining IRF1 at each post CHX time points between WT and mutant IRF1.

B. Half-lives of IRF1 protein expressed in MRC5 or HEK293 cells determined by CHX assays related to Fig 6A and 6B
C. Half-lives in MRC5 and HEK293 related to Fig 6C and 6D
D. CHX chase in MRC5 cells related to figure 6D but with the addition of the T181A mutant
E. CHX chase in MRC5 cells (expressing WT FLAG-IRF1) pre-treated with the GSK3 inhibitor X / BIO (1 µM) for 1 hour prior to CHX addition and chase as before.

Supplementary Figure 4. Fbxw7α interacts with IRF1

A. HEK293 transfected with GST or GST-Fbxw7 (α, β, γ) expression plasmids and FLAG-IRF1 for 48 hours. 6 hours prior to lysis cells were treated with MG132 (10 µM). Lysates were incubated with Glutathione-Sepharose beads for 3 hours. Captured proteins were revealed by immunoblot with anti-GST and anti-FLAG antibodies. 10% inputs demonstrate expression of transfected proteins.
B. Indirect immunofluorescence of GSK3β and FBXW7 in Cos7. Scale bar = 10µm.

Supplementary Figure 5. Fbxw7α regulates IRF1 ubiquitination, half-life and transcriptional activity

A. HEK293 CHX chase of cells expressing FLAG-IRF1, HA-Fbxw7α FL or HA-Fbxw7α ΔWD40.
B. Lysates related to S5A, note the ΔWD40 mutant migrates below the nonspecific band detected by the HA antibody.
C. Western blot panel related to figure 10A
D. Ubiquitination of IRF1 K→R mutants in HEK293 co-transfected with Fbxw7. Lysates were enriched for ubiquitinated proteins by Ni2+ pulldown and probed with FLAG antibody. Inputs show expression of transfected proteins.
E. Quantification of ubiquitination status of IRF1 K→R mutants using indicated ubiquitin variants. MRC5 transfected with indicated IRF1 and Ub mutants were assayed as before. Data is for three independent repeats. .
F. Luciferase reporter assays in MRC5 cells expressing indicated IRF1 construct and the 4XISRE-Luc reporter construct.

Supplementary Figure 6. Thr^{181} and Fbxw7 are required for IRF1 anti-proliferative activity in cancer cells.

A. Bar graph of data presented in 11C. Data is shown as % change in cell number where empty vector is expressed as 100%. Error bars = SEM.

Supplementary Figures

Supplementary Figure 1 IRF1 is phosphorylated by GSK3β

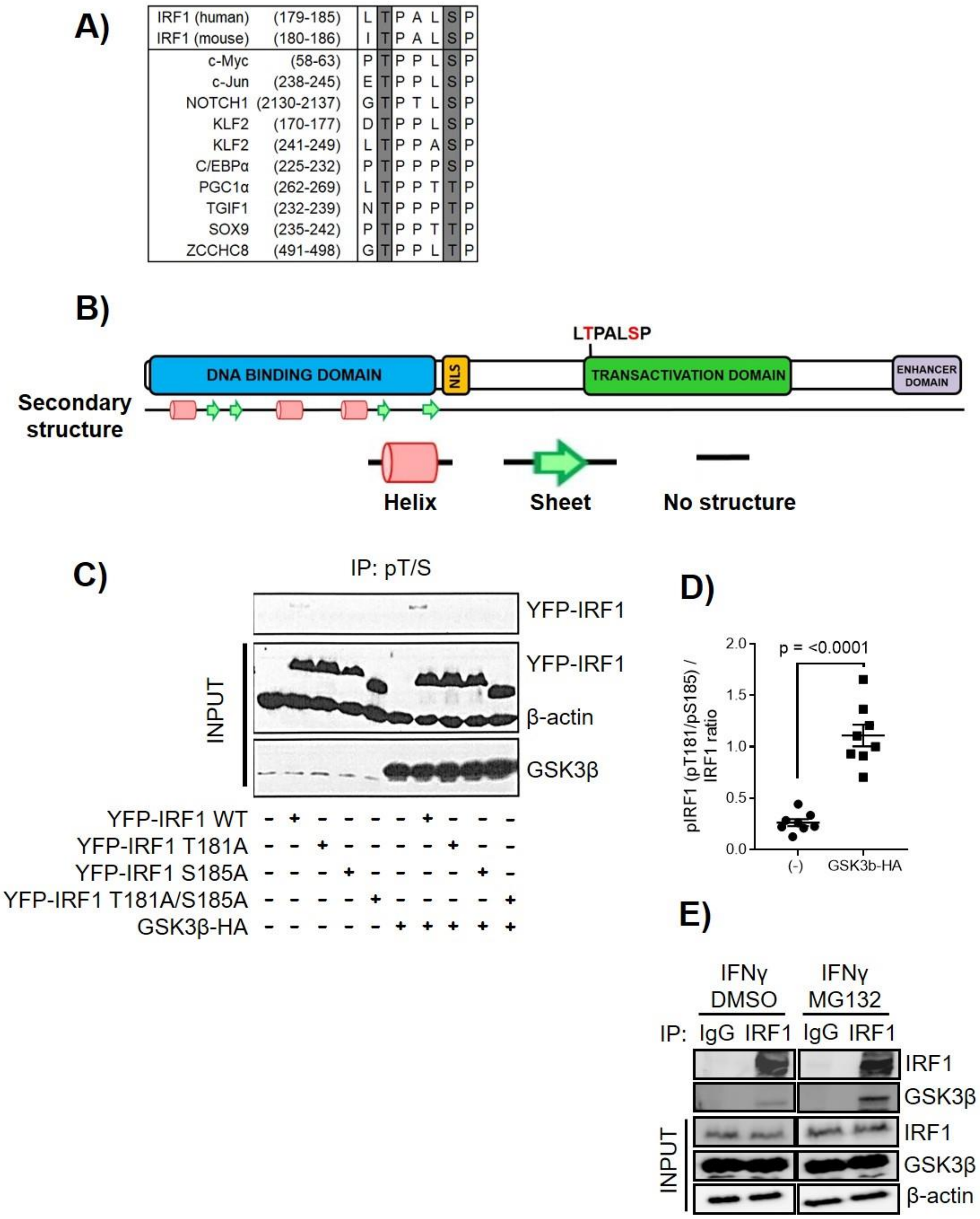

Supplementary Figure 2. GSK3β is required for IRF1 transcriptional activity

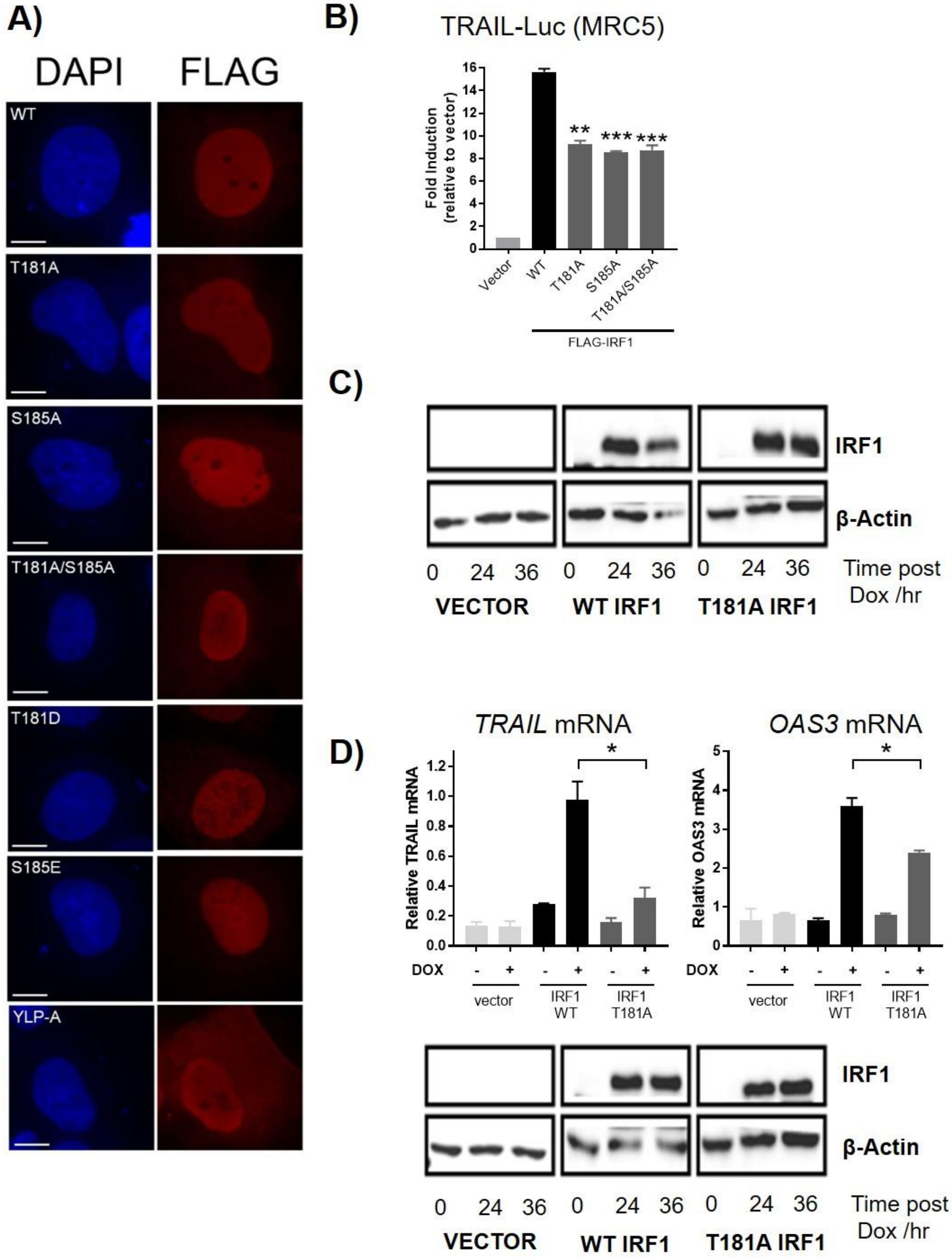

Supplementary Figure 2. GSK3β is required for IRF1 transcriptional activity

E)

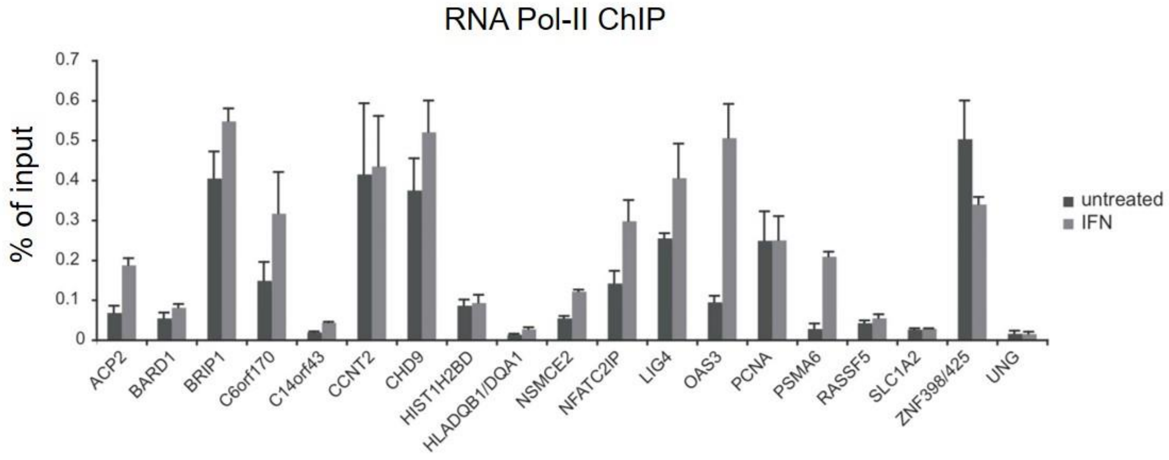

H3396 cells

F)

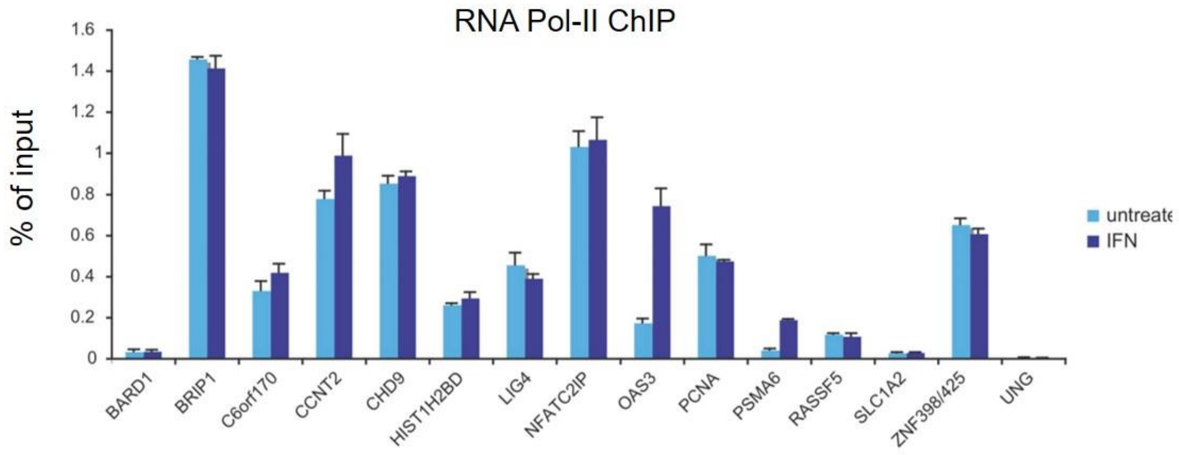

MRC-5 cells

Supplementary Figure 3. Stability of IRF1 T181A is not affected by GSK3β over-expression and GSK3β inhibition stabilises IRF1

A)

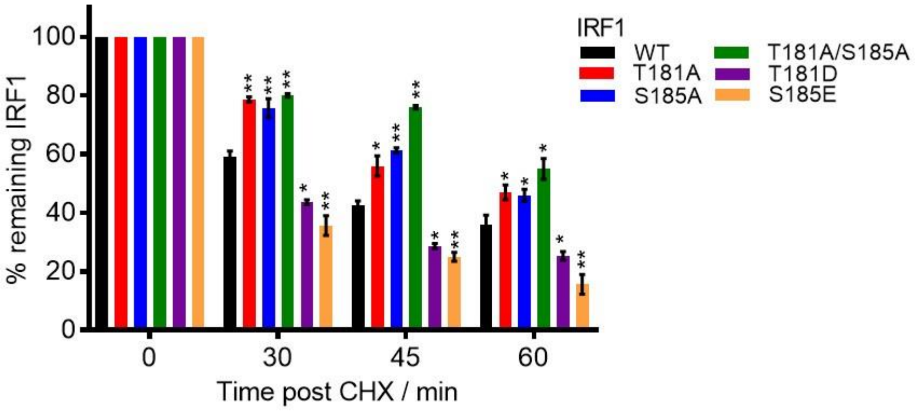

B)

	Half life / minutes	
	**HEK293**	**MRC5**
**WT**	32	35
**T181A**	60	70
**S185A**	50	55
**TS-A**	65	75
**T181D**	21	22
**S185E**	22	23

C)

	Half life / minutes	
**IRF1 +**	**HEK293 exogenous mouse IRF1**	**MRC5 endogenous human IRF1**
**vector**	45	40
**GSK3β WT**	25	30
**GSK3β K85A**	80	70

D)

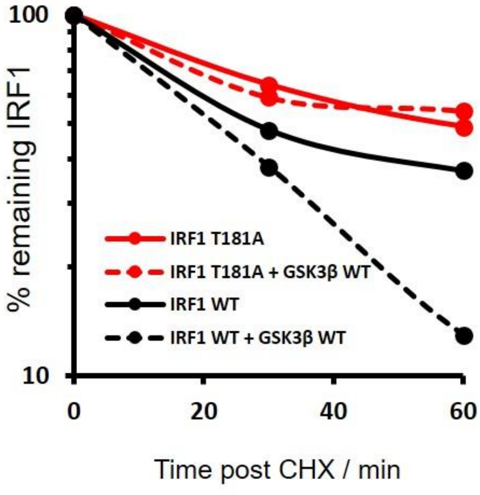

E)

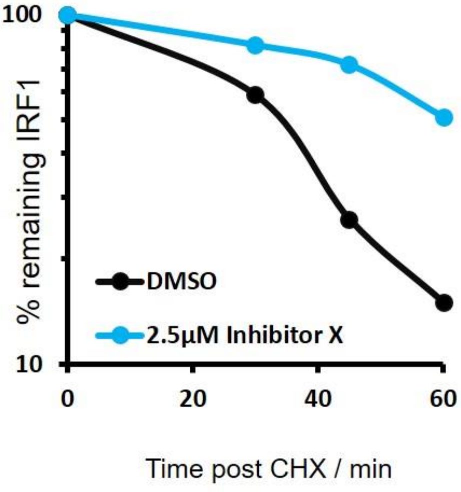

Supplementary Figure 4. Fbxw7α interacts with IRF1

A)

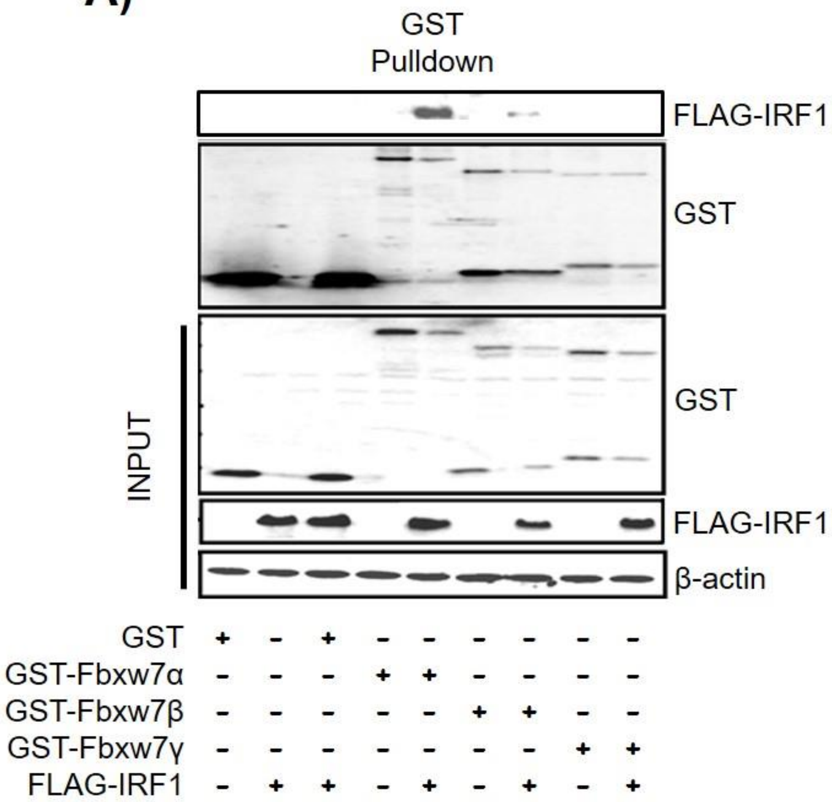

B)

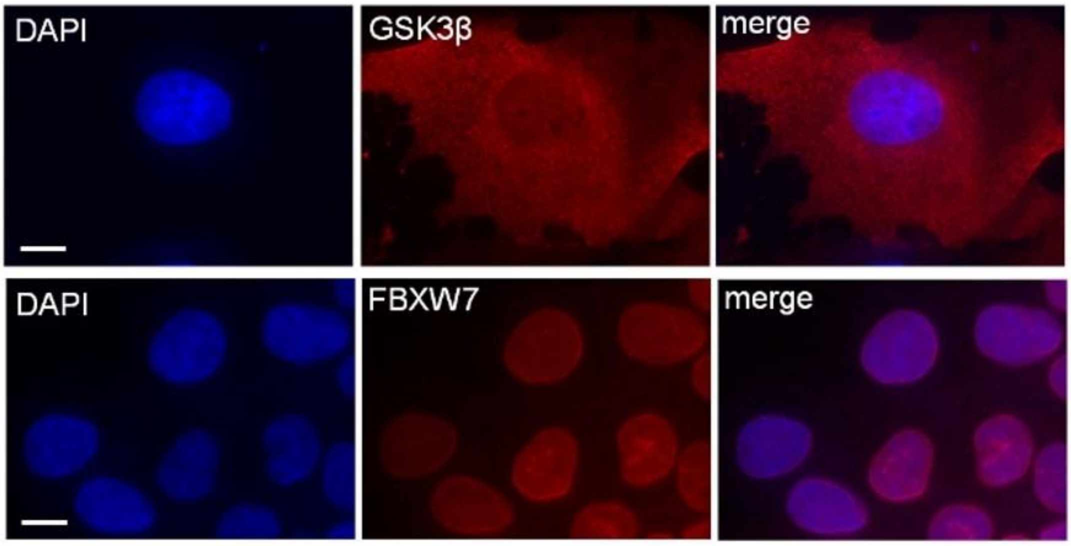

Supplementary Figure 5. Fbxw7α regulates IRF1 ubiquitination, half-life and transcriptional activity

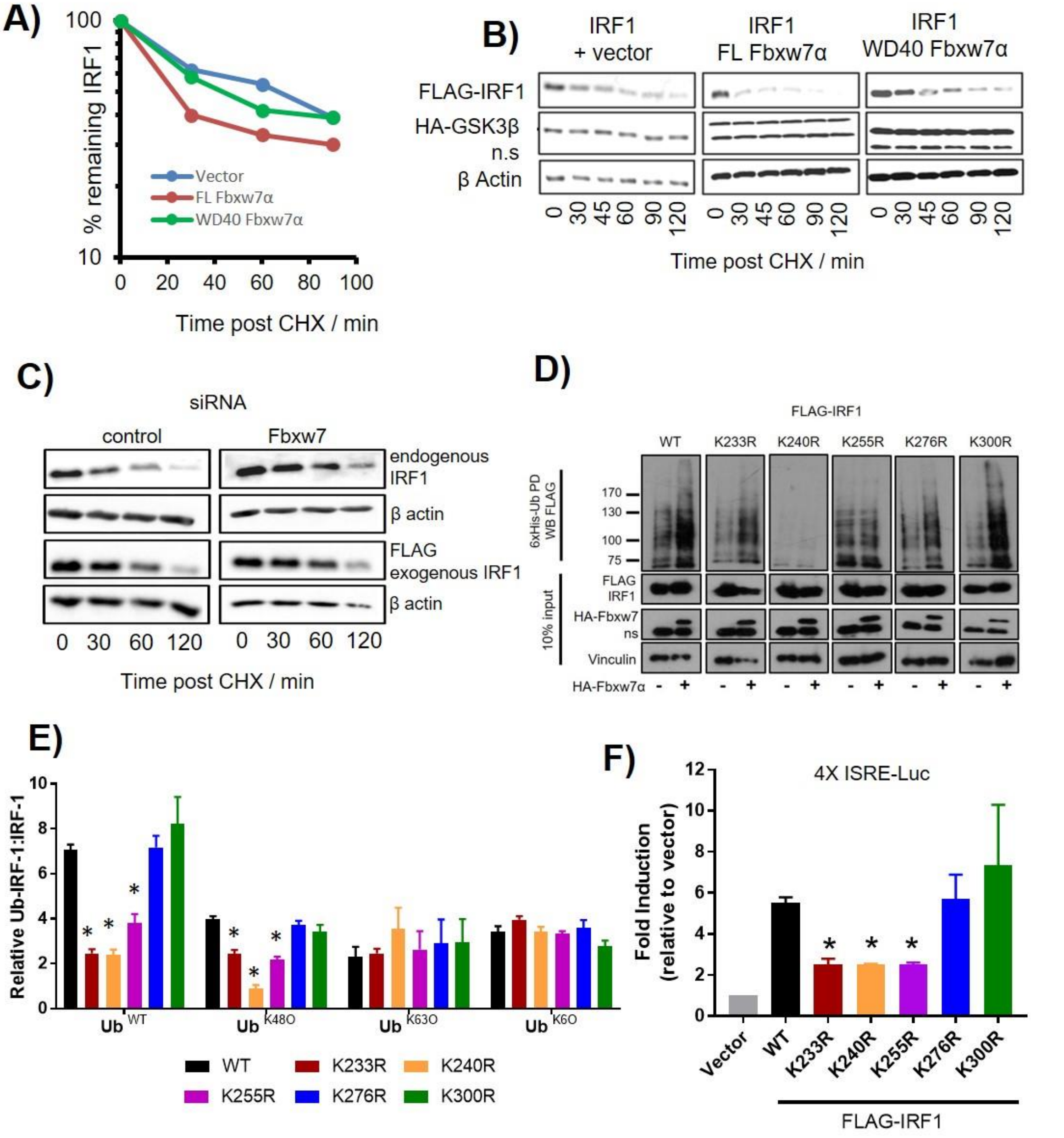

Supplementary Figure 6 Thr^{181} and Fbxw7 are required for IRF1 anti-proliferative activity in cancer cells.

A)

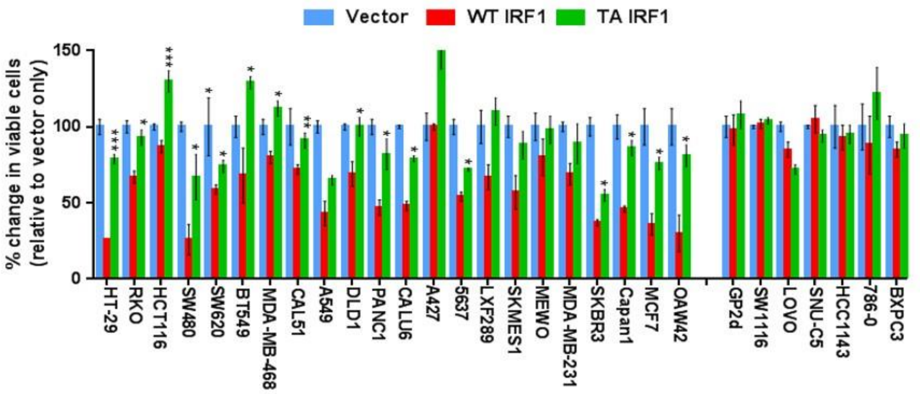

Supplementary Table 1. Cell lines

Cell Line	Tissue	Growth medium	Fbxw7 status*
5637	Urinary Bladder	RPMI	WT
A427	Lung	EMEM	WT
A549	Lung	DMEM	WT
BT549	Breast	DMEM	WT
CAL51	Breast	DMEM	WT
CAPAN1	Pancreas	DMEM	WT
CALU6	Lung	EMEM	WT
DLD1	Colon	DMEM	WT
HCT116	Colon	DMEM	WT
HT29	Colon	DMEM	WT
LXF289	Lung	RPMI	WT
MCF7	Breast	DMEM	WT
MDA-MB-231	Breast	DMEM	WT
MDA-MB-468	Breast	DMEM	WT
MeWo	Melanoma	EMEM	WT
OAW42	Ovarian	DMEM	WT
PANC1	Pancreas	DMEM	WT
RKO	Colon	DMEM	WT
SKBR3	Breast	RPMI	WT
SKMES1	Lung	EMEM	WT
SW480	Colon	DMEM	WT
SW620	Colon	DMEM	WT
786-O	Kidney	RPMI	Homozygous deletion
BxPC3	Pancreas	RPMI	Homozygous deletion
GP2d	Colon	DMEM	H580R (WD40 repeats)
HCC1143	Breast	RPMI	Deletion (Mao *et al.* 2010)
LoVo	Colon	DMEM	R505C (WD40 repeats)
SNU-C5	Colon	DMEM	S668fs39
SW1116	Colon	DMEM	H460Y (WD40 repeats)
H3396	Breast	RPMI	WT
MRC5	Lung fibroblast	Alpha MEM	WT

* Fbxw7 status was determined from the cancer cell line encyclopaedia

Supplementary Table 2. Antibodies

**Antibody / Concentration**	**Supplier**	**Use**
Murine IRF1 (M20) (1:1000)	Santa Cruz	WB, IP
Human IRF1 (C20) (1:1000)	Santa Cruz	WB, IP
Phospho-c-Myc (Thr^{58}/Ser^{62}) (pT/S) (1:1000)	Santa Cruz	WB, IP
GSK3β (27C10) (1:2000)<br>GSK3β ab93926 (1:500)	CST<br>Abcam	WB, IP<br>IF
Phospho-Threonine-Proline (9391) (1:1000)	CST	IP
GFP (1:2000)	Roche	WB
GST (1:2000)	SIGMA	WB
FLAG M2 (1:2000)	SIGMA	WB, IP
HA 12CA5 (1:2000)	SIGMA	WB, IP
β-Actin (A5441) (1:2000)	SIGMA	WB
Tubulin (T8203) (1:2000)	SIGMA	WB
RNAP II (N20)	Santa Cruz	ChIP
pSerine 2 RNAP II (ab5095)	Abcam	ChIP
Vinculin (ab129002) (1:2000)	Abcam	WB
Ki57 (ab15580)	Abcam	IF
Histone H3 (ab10779) (1:2000)	Abcam	WB
GAPDH (ab8245) (1:2000)	Abcam	WB
Fbxw7 (ab109617) (1:1000)	Abcam	IF
Lamin B1 (ab16048) (1:2000)	Abcam	WB

Supplementary table 3. Primers

Primer	Sequence
IRF1 T181A	F: ATGGAAAGGGACATAGCTCCAGCACTGTCACCG<br>R: CGGTGACAGTGCTGGAGCTATGTCCCTTTCCAT
IRF1 S185A	F: CATAACTCCAGCACTGACACCGTGTGTCGTCAG<br>R: CTGACGACACACGGTGTCAGTGCTGGAGTTATG
IRF1 T181A/S185A	F: GGAAAGGGACATAGCTCCAGCACTGGC<br>R: GCCAGTGCTGGAGCTATGTCCCTTTCC
IRF1 T181D	F: GGACTTGGATAGGAAAGGGACATAGATCCAGCACTGTCA<br>R: TGACAGTGCTGGATCTATGTCCCTTTCCATATCCAAGTCC
IRF1 S185E	F: AGGGACATAACTCCAGCACTGGAGCCGTGTGTCGTCAGCAGCAGT<br>R: TCCCTGTATTGAGGTCGTGACCTCGGCACACAGCAGTCGTCGTCA
IRF1 YLP-A	F: GCGGGTGGCCCGGATGGCGGCACCCCTCACCAGG<br>R: CCTGGTGAGGGGTGCCGCCATCCGGGCCACCCGC
IRF1 K233R	F: GGATGAGGAAGGGAGGATAGCCGAAGACC<br>R: GGTCTTCGGCTATCCTCCCTTCCTCATCC
IRF1 K240R	F: GATAGCCGAAGACCTTATGAAGGCTCTTTGAACAGTCTGAG<br>R: CTCAGACTGTTCAAAGAGCCTCATAAGGTCTTCGGCTATC
IRF1 K255R	F: GACACACATCGATGGCAGGGGATACTTGCTCAATG<br>R: CATTGAGCAAGTATCCCCTGCCATCGATGTGTGTC
IRF1 K276R	F: GGAGACTTCAGCTGCAGAGAGGAACCAGAGATTG<br>R: CAATCTCTGGTTCCTCTCTGCAGCTGAAGTCTCC
IRF1 K300R	F: CATGTCTTCACGGAGATGAGGAATATGGACTCCATCATG<br>R: CATGATGGAGTCCATATTCCTCATCTCCGTGAAGACATG
IRF1 (pEYFP)	F: ATAATAAGATCTATGCCAATCACTCGAATG<br>R: ATAATATCTAGACTATGGACAAGGAAT
IRF1 (FLAG)	F: ATAATAAAGCTTATGCCAATCACTCGAATG<br>R: ATAATATCTAGACTATGGACAAGGAAT
HA-Fbxw7α FL	F: ATAATAGAATTCATGAATCAGGAACTGCTCTCTGTG<br>R: TATTATTCTAGATCACTTCATGTCCACATCAAAGTC
HA-Fbxw7α FL ΔWD40	F: ATAATAGAACCTAAGGTGCTGAAAGGACATGAT<br>R: TATTATTCTAGATCAAGATTTGAGTTCTCCTCGCCT
RT-QPCR primers	Clarke *et al.* 2004
ChIP primers	Clarke *et al.* 2004

1. Frontini, M., Vijayakumar, M., Garvin, A. and Clarke, N. (2009) A ChIPchip approach reveals a novel role for transcription factor IRF1 in the DNA damage response. *Nucleic Acids Research*, **37**, 1073-1085.
